# Machine Learning-Based Genome-Wide Salivary DNA Methylation Analysis for Identification of Noninvasive Biomarkers in Oral Cancer Diagnosis

**DOI:** 10.3390/cancers14194935

**Published:** 2022-10-08

**Authors:** John Adeoye, Chi Ching Joan Wan, Li-Wu Zheng, Peter Thomson, Siu-Wai Choi, Yu-Xiong Su

**Affiliations:** 1 Division of Oral and Maxillofacial Surgery, Faculty of Dentistry, The University of Hong Kong, Hong Kong 999077, China; 2 College of Medicine and Dentistry, James Cook University, Cairns, QLD 4870, Australia

**Keywords:** biomarkers, diagnosis, DNA methylation, epigenomics, oral cancer, oral potentially malignant disorders

## Abstract

**Simple Summary:**

Because tissue biopsy is the gold standard for diagnosing oral cancer, it is often performed to confirm disease during screening, management, and monitoring. However, many reports are negative. Salivary biomarkers can provide the preliminary stratification of suspicious lesions to encourage patient selection in clinical practice. However, the discovery and implementation of salivary biomarkers still need to be refined. Therefore, in this study, we successfully utilized machine learning techniques to select optimal methylome biomarkers that may be applied for oral cancer diagnoses.

**Abstract:**

This study aims to examine the feasibility of ML-assisted salivary-liquid-biopsy platforms using genome-wide methylation analysis at the base-pair and regional resolution for delineating oral squamous cell carcinoma (OSCC) and oral potentially malignant disorders (OPMDs). A nested cohort of patients with OSCC and OPMDs was randomly selected from among patients with oral mucosal diseases. Saliva samples were collected, and DNA extracted from cell pellets was processed for reduced-representation bisulfite sequencing. Reads with a minimum of 10× coverage were used to identify differentially methylated CpG sites (DMCs) and 100 bp regions (DMRs). The performance of eight ML models and three feature-selection methods (ANOVA, MRMR, and LASSO) were then compared to determine the optimal biomarker models based on DMCs and DMRs. A total of 1745 DMCs and 105 DMRs were identified for detecting OSCC. The proportion of hypomethylated and hypermethylated DMCs was similar (51% vs. 49%), while most DMRs were hypermethylated (62.9%). Furthermore, more DMRs than DMCs were annotated to promoter regions (36% vs. 16%) and more DMCs than DMRs were annotated to intergenic regions (50% vs. 36%). Of all the ML models compared, the linear SVM model based on 11 optimal DMRs selected by LASSO had a perfect AUC, recall, specificity, and calibration (1.00) for OSCC detection. Overall, genome-wide DNA methylation techniques can be applied directly to saliva samples for biomarker discovery and ML-based platforms may be useful in stratifying OSCC during disease screening and monitoring.

## 1. Introduction

Oral cancer is the most common head and neck malignancy and accounts for about 2.3% of cancer-related deaths worldwide [1]. The early detection of primary tumors and tumor recurrence is central to obtaining a better disease-specific and overall prognosis [2,3,4,5]. Currently, tumor diagnoses before intervention and during disease monitoring are reliant on the performance of a tissue biopsy on and the histopathology of apparent lesions. However, concerns regarding the representativeness of sampled tissues for an optimal diagnosis, postoperative complications, and potential tumor seeding still exist [6,7]. During disease monitoring, patients are subjected to multiple biopsies for lesions emanating in or around tumor resection sites, which are often false positives [6,8]. Likewise, for organized/opportunistic oral cancer screenings, the reduced compliance of patients with suspicious diseases that are identified has been linked to their concerns regarding the need for and immediate post-operative complications of tissue biopsy. Therefore, utilizing accurate and specific platforms for delineating high-risk diseases before performing a tissue biopsy has the potential to improve the impact and efficiency of oral-cancer-screening programs by allaying patient concerns about the importance of the procedure according to this preliminary test [8,9].

Liquid biopsy, especially involving saliva and mouth-rinse samples, has the potential to circumvent the problems posed by tissue biopsies [10,11]. Explorations into the use of salivary molecular markers that are specific to or preferential for oral cancer have suggested potential candidates for clinical applications, with transcriptomic and epigenomic markers being the most promising [12,13,14,15,16]. Furthermore, for epigenetic markers, our group and others have reported a better diagnostic accuracy when biomarker panels were used as opposed to single biomarkers [9,17,18]. However, it was observed that true genome-wide techniques, such as whole-genome bisulfite sequencing (WGBS) and reduced-representation bisulfite sequencing (RRBS), were not being utilized during methylome biomarker discovery, which may have limited the optimal biomarker selection [9,13]. Moreover, methods to operationalize DNA methylation markers in clinical settings were not being suggested or utilized in the available reports [8,9,19].

Machine learning (ML) platforms are increasingly being implemented with diverse feature sets for the accurate diagnosis of different malignancies [20,21]. These intelligent models have been found to have higher performances than alternate methods for the construction of assistive diagnostic platforms and, in many cases, they have been found to have an equivalent or slightly reduced performance when compared to clinicians [22,23,24]. Therefore, integrating ML and salivary biopsy as an ML-assisted liquid-biopsy platform could potentially optimize the performance of the salivary biomarkers in diagnosing oral cancer. This may also provide an avenue to operationalize and validate salivary biomarkers objectively and efficiently. To this end, this study examined the feasibility of utilizing a scalable ML-based platform for biomarker selection and the noninvasive diagnosis of oral cancer using salivary methylome biomarkers. This study hypothesized that biomarkers identified by genome-wide methylation analysis techniques like RRBS using saliva samples can efficiently distinguish patients with oral cancer from patients with oral potentially malignant disorders (OPMDs) or oral mucosal diseases. This could be beneficial as a tool for the early diagnosis of oral cancer during disease screening, indicating tumor remission during treatment, and the timely detection of tumor recurrence during disease surveillance.

## 2. Materials and Methods

### 2.1. Patients and Eligibility Criteria

Patients with oral squamous cell carcinoma (OSCC) and OPMDs were enrolled in a study to assess and validate methylome markers in their saliva for cancer diagnoses. This nested cohort comprised patients who either presented to our institution or were referred through an oral-cancer-community screening program conducted by the authors [25]. Participant recruitment was carried out from June 2020 to January 2022. Only patients above 18 years old with primary untreated OSCC and OPMDs were included in this study. Patients with previous head and neck malignancies, radiation therapy, and histologic variants other than squamous cell carcinomas, such as oral melanomas and sarcomas, were not considered. Additionally, patients with severe debilitating and inflammatory conditions were not included. The scope of OSCC was according to the International Classification of Disease (ICD) codes C00.3–C00.6, C02–C04, C05.0, and C06 to include only tumors affecting the oral cavity following Montero et al. [26] and Conway et al. [27]. Meanwhile, OPMDs were defined according to the recommendations by the WHO Collaboration Centre for oral cancer in 2020 [28]. The patients were enrolled using the prospective-specimen-collection, retrospective-blinded-evaluation (PRoBE) protocol, and the identification of outcome status for the first 50 patients was conducted for biomarker discovery and ML analysis [19,29,30,31]. From this patient pool, 33 patients, comprising patients with OSCC and OPMDs, were randomly selected using computer-generated numbers for reduced-representation bisulfite sequencing analysis. Demographical, clinical, and pathological characteristics of the patient cohort are detailed in Table 1. OPMD subtypes included oral lichen planus (43.8%), erythroplakia (12.6%), erythroleukoplakia (6.3%), leukoplakia (31.3%), and oral submucous fibrosis (6.3%).

### 2.2. Oral Rinse Collection and DNA Extraction

Mouth rinses were collected following histologic confirmation of the disease and before treatment commencement. This was performed at least one hour after the patient’s last meal. Patients were asked to pre-rinse for 30 s with water or chlorhexidine and wait for 5 min before sampling was conducted using 20 mL of PBS. Rinsing was then performed for 1 min and emptied into a 50 mL falcon tube on ice. The samples were stored at 20 °C until further processing was conducted within 24 h. Afterward, the collected samples were centrifuged at 2000 rpm for 10 min to isolate the cell pellets. Genomic DNA extraction was performed using the QIAamp DNA Blood Mini kit (QIAGEN, Hilden, Germany) according to the manufacturer’s protocol for saliva. DNA quantity was assessed using both spectrophotometric and fluorometric methods, and the integrity was checked on 0.8% and 2% agarose gel electrophoreses.

### 2.3. Reduced-Representation Bisulfite Sequencing (RRBS)

A library was constructed on the protocol of the Premium RRBS kit (Diagenode S.A, Denville, NJ, USA). A total of 100 ng of genomic DNA from each sample was used for library construction. Samples were digested with the MspI restriction enzyme overnight, after which the fragmented DNA was end-repaired. Poly-A-tailing of the 3′ ends and indexed adaptor ligation was performed afterward. Fragment size selection was conducted using AMPure XP Beads, which was followed by qPCR quantification and sample pooling (same amount for all samples) using the Ct values obtained. Bisulfite conversion of the sample pool to convert unmethylated cytosine to thymine was then performed overnight. Another round of qPCR quantification was conducted to determine the optimal cycle number needed for enrichment, after which the DNA fragments were then amplified by 14 cycles during library enrichment. The quality control of the enriched library was performed using a Bioanalyzer (Agilent Technologies, Santa Clara, CA, USA), Qubit fluorometric analysis, and qPCR. The generated library was sequenced using the 151 paired-end protocol of the NovaSeq 6000 system (Illumina, San Diego, CA, USA).

### 2.4. Sequence Alignment and Differential Methylation Analysis

The quality control and adapter trimming of raw reads were conducted using the *Trim Galore* program (Phred score ≥ 20), while the alignment of filtered reads to the hg38 reference genome and calling of methylated cytosine followed, using *Bismark* software [32]. *Methylkit* [33] was used for differential methylation analysis. Bismark coverage files were entered into the software, and CpG sites common to all samples with a minimum of 10× coverage were merged. Small-sized differentially methylated regions (DMRs) with read lengths amenable to subsequent pyrosequencing verification were the unit of regional analysis performed. A total of 100 bp regions that covered a minimum of ten CpG sites were tiled with a step size of 25. Logistic regression analysis was performed for each CpG site and 100 bp region based on the disease groups i.e., OSCC and OPMDs. False discovery rates were controlled by transforming the *p*-values to *q*-values using the sliding-linear-model (SLIM) method [34]. Differentially methylated CpG sites (DMCs) and DMRs were selected according to a mean methylation percent difference ≥ 5 (using the read coverage for weighting) and *q*-values < 0.01. The functional annotation of DMCs and DMRs based on their locations, relative to genes and CpG islands, was conducted using *Genomation* and Hypergeometric Optimization of Motif Enrichment (*HOMER)* software for the description of these sites. DMRs annotated to genes were enriched according to gene ontology (GO) terms, based on the biological process and KEGG pathways using the *clusterProfiler* program [35].

### 2.5. Machine Learning Models

Percent methylation of both DMCs and DMRs across the different annotated regions and samples was utilized to construct ML models for the classification of OSCC from OPMDs. Machine learning models implemented for disease classification included the linear and radial basis function (RBF), support vector machines (SVM), adaptive boosting (AdaBoost), k-nearest neighbors (kNN), random forest (RF), decision tree (DT), extremely randomized trees (ExtraTrees), and gradient boosting machines (GBM). Each DMC/DMR was utilized as a predictive feature in an initial ML model for all techniques. Due to the high dimensionality of the data, feature selection was also performed to foster the clinical application of these methylome biomarkers. Three robust feature-selection techniques, including the analysis of variance (ANOVA) F-statistic, minimum redundancy–maximum relevance (MRMR), and least absolute shrinkage and selection operator (LASSO), were used to select different biomarker combinations for ML modeling. ANOVA and MRMR are two filter-based feature-selection techniques based on univariate and multivariate analysis, respectively, while LASSO is an embedded feature-selection method used over regression analysis for regularization [36]. As wrapper-based feature-selection methods are known to be model-dependent with the potential for different optimal feature sets according to the ML techniques, these methods were not considered in this study.

Models based on DMCs and DMRs selected by the three feature-selection methods were also constructed. Afterward, the concordance of the selected methylome biomarkers for all three feature-selection methods was assessed, and these consensus biomarkers (DMCs and DMRs) were implemented with three outperforming ML algorithms to assess their performance. Model training and validation were implemented using leave-one-out cross-validation as the data resampling method. In all, the performance of 70 classification models was compared. The performance metrics used for model selection included the recall, specificity, precision, overall accuracy, area under the receiver operating characteristic curve (AUC), and calibration (O/E). Additionally, global Shapley additive explanations (SHAP) values were obtained for the optimal ML models based on DMCs and DMRs to explain their rationale for the predicted classes (OSCC vs. OPMDs) as a function of the predictors.

### 2.6. Computation

Descriptive statistics were performed using SPSS v 27. Bioinformatics analyses and machine learning analyses were implemented in R statistical software v 4.1.0 and Python v 3.7.6 (Python Software Foundation, Wilmington, DE, USA). The reporting of this study was conducted according to the Transparent Reporting of a multivariable prediction model for individual prognosis or diagnosis (TRIPOD) statement [37].

## 3. Results

### 3.1. Sequencing Quality and Descriptive Analysis

Excellent bisulfite conversion was recorded with an estimated rate of at least 99% for all samples. Reads were assigned to individual samples with an average of 88% of the bases with a Q30 Phred score and an average of 47.3% of read pairs that were uniquely mapped to the reference genome. Three-dimensional plots showing the distribution of methylation percentages based on sequencing reads with 10× coverage for patients in the OSCC and OPMD cohorts are displayed in Figure S1. These showed that, for the majority of the CpG site, percent methylation was either below 5% or above 95%. Also, histogram of CpG read coverage per base for each sample is deposited in File S1.

### 3.2. Base-Pair Resolution Analysis

**Figure 1:**
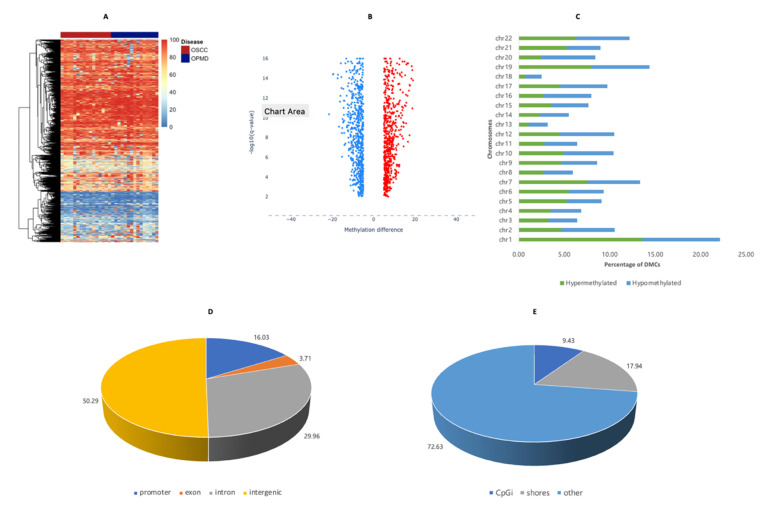
Base-pair resolution analysis for identification and description of DMCs. (**A**) Heatmap comprising the methylation percentages of differential CpG sites for all samples (**B**) Volcano plot of weighted mean methylation difference for hypermethylated (red) and hypomethylated (blue) DMCs (**C**) Autosomal annotation of DMCs (**D**) Genomic annotation of DMCs (**E**) CpG island annotation of DMCs.

In total, 1745 DMCs were identified for OSCC relative to other mucosal diseases in the OPMDs group. A heatmap of all DMCs and their methylation percentages is shown in Figure 1A. Additionally, the mean methylated percentage differences for all DMCs and their corresponding *q*-values are presented in Figure 1B, with 854 CpG sites differentially hypermethylated while 891 CpG sites were hypomethylated. The distribution of the DMCs per autosome is displayed in Figure 1C with a higher proportion of aberrantly methylated CpG sites being mapped to the first chromosome than others. An annotation of the DMCs based on genomic features found that a majority of the DMCs were mostly enriched in the intergenic regions with about 16% mapped to promoter sites (Figure 1D). Further annotation showed that 9.4% of DMCs were within CpG islands, while 17.9% were within CpG island shores which, in this study, represented a 2kb-long region on both sides of the CpG islands (Figure 1E). As for all DMCs, a similar distribution in the genomic and CpG island annotations was observed when stratified by hypermethylated and hypomethylated DMCs.

**Figure 2:**
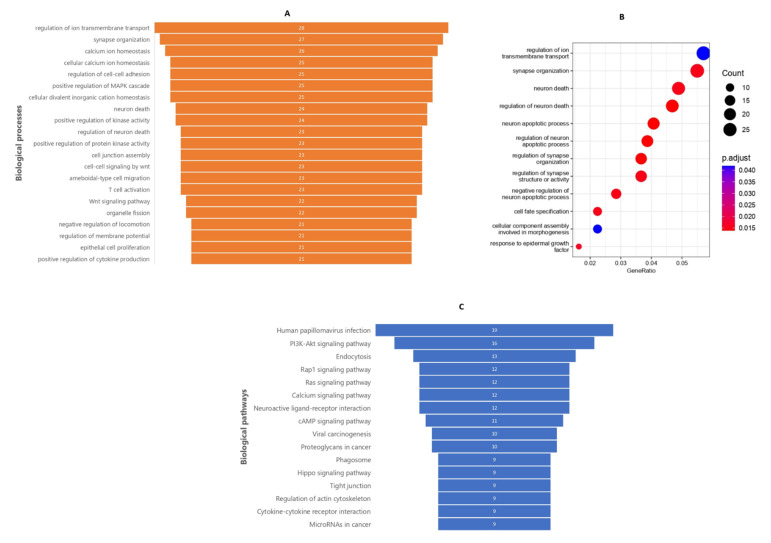
Gene ontology (GO-BP) and KEGG pathway analysis enrichment of genes associated with DMCs. (**A**) List of biological processes based on the DMC count after GO-BP analysis. (**B**) List of significant biological processes associated with DMC genes after GO-BP analysis. (**C**) List of biological pathways enriched for DMC-associated genes.

To map DMCs to pathways in which they were enriched, gene ontology was performed according to biological processes (GO-BP) using 1283 DMCs annotated to 750 genes. The enriched DMC set based on their count, irrespective of statistical significance, is listed in Figure 2A, which alluded to common deregulated processes in tumors. The enriched significant biological processes included ion transport, regulation of apoptosis, synapse organization, cellular differentiation, and the response to epidermal growth factor (EGF) (Figure 2B). KEGG pathway analysis also identified cancer-related pathways in which the DMCs were enriched (Figure 2C) with the genes associated with these DMCs and their network, which is shown in Figure S2. The four most-implicated pathways included the PI3K-Akt, Endocytosis, Rap1, and Ras pathways.

### 3.3. Machine-Learning-Based Optimal DMC Selection and Disease Prediction

**Figure 3:**
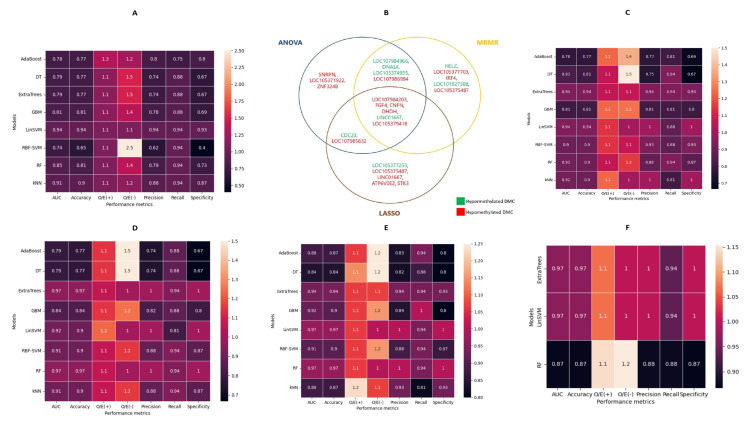
Performance of machine learning models for predicting OSCC using DMC as features. (**A**) Initial models comprising all 1745 DMCs. (**B**) Selected features by the three feature-selection methods and their concordance. (**C**) Machine learning models based on ANOVA-selected DMC sets for predicting OSCC. (**D**) Machine learning models based on MRMR-selected DMC sets for predicting OSCC. (**E**) Machine learning models based on LASSO-selected DMC sets for predicting OSCC. (**F**) Machine learning models based on six consensual DMCs for predicting OSCC.

The methylation percentages for all 1745 DMCs were extracted and used to develop predictive models for the diagnosis of OSCC among patients with suspicious oral mucosal diseases. The performance of different ML classifiers based on all 1745 predictive features is shown in Figure 3A. The linear SVM model had the best recall, specificity, precision, and O/E calibration of 0.94, 0.93, 0.94, and 1.1 for OSCC prediction, while the worst discrimination and calibration estimates were obtained for SVM with RBF kernel.

To reduce model overfitting and enhance feasibility, three feature-selection techniques (ANOVA, MRMR, and LASSO) were compared to select an optimal set of DMCs that could be clinically utilized for noninvasive OSCC detection. The aberrant CpG sites chosen using the different techniques and their interactions are listed in Figure 3B. Fifteen optimal DMCs were selected using ANOVA and MRMR, while LASSO selected 13 optimal DMCs. Across all three feature-selection methods, six consensual DMCs were selected, while six DMCs in total were also selected by any two methods (Table S1).

The performance of the ML models for predicting OSCC occurrence according to the different DMC sets is shown in Figure 3C–E. Using the ANOVA-selected DMCs, linear SVM and ExtraTrees models had the highest AUC and accuracy of 0.94, although the linear SVM had a higher specificity (1.00) while ExtraTrees had a higher sensitivity (0.94). Compared to the ANOVA-selected DMCs, optimal aberrant CPG sites selected by MRMR performed better: RF and ExtraTrees had an AUC, recall, and specificity of 0.97, 0.94, and 1.00. Both linear SVM and RF performed best using the LASSO-based DMCs and matched the highest performances on the MRMR-based DMC set with an AUC, recall, and specificity of 0.97, 0.94, and 1.00.

As linear SVM, ExtraTrees, and RF were the outperforming ML models, the optimal set of six DMCs selected by all feature-selection techniques were used to classify OSCC and OPMDs. Linear SVM and ExtraTrees both had similar performance metrics (AUC; 0.97; recall: 0.94; specificity: 1.00) which was higher than those of RF (AUC; 0.87; recall: 0.88; specificity: 0.87). Additionally, we evaluated the effect of including demographic variables in Table 1 to the models; however, this did not improve or degrade the performance of the models. SHAP values indicative of model explainability for the predicted outputs by the linear SVM and ExtraTrees classifiers are displayed in Figure S3. Overall, the difference in the methylation percentages based on DMC hypomethylation of *FGF4* was the most important feature for the delineation of OSCC and OPMDs by both algorithms.

### 3.4. Differentially Methylated Region (DMR) Analysis

**Figure 4:**
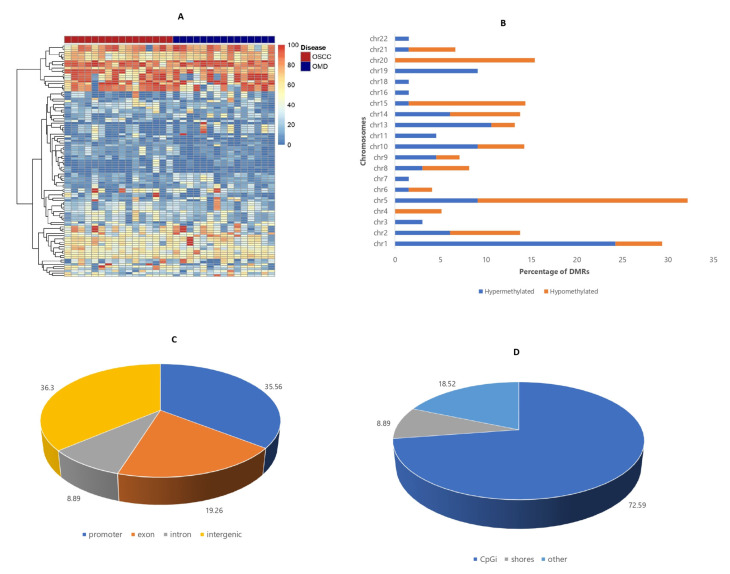
100 bp regional methylation analysis for description of DMRs. (**A**) Heatmap comprising the methylation percentages of aberrantly methylated regions in all samples. (**B**) Autosomal annotation of DMCs. (**C**) Genomic annotation of DMRs. (**D**) CpG island annotation of DMRs.

Differential analysis was also conducted according to 100 bp genome regions comprising at least 10 CpG sites as a function of the proportion of the methylated and total reads summed across all sites. The analysis found a total of 105 DMRs in OSCC patients with 62.9% being hypermethylated regions and 37.1% being hypomethylated regions. Heatmap of all DMRs is displayed in Figure 4A. Autosomal annotations of the DMRs are also shown in Figure 4B with a higher proportion of the hypermethylated DMRs found on chromosome 1 and the hypomethylated DMRs found on chromosome 5. In contrast to functional annotation obtained for DMCs, a similar proportion of DMRs were enriched in intergenic (36.3%) and promoter regions (35.56%) (Figure 4C). In addition, most hypermethylated DMRs were annotated to promoter than intergenic regions (42.03% vs. 23.19%) while most hypomethylated DMRs were enriched in the intergenic than promoter region (50% vs. 28.79%). CpG island annotation was also performed with 72.59% located within islands than shores (8.89%) (Figure 4D). Additionally, 82.61% of hypermethylated DMRs and 62.12% of hypomethylated DMRs were annotated to CpG islands.

According to GO-BP analysis based on 84 DMRs enriched in 47 genes, the majority of the genes associated with the DMRs were enriched in processes involving cell-cell adhesion, chromatin organization, and pattern specification (Figure S4). Likewise, KEGG pathway analysis identified five DMR-associated genes that were involved in different pathways also shown in Figure S4.

### 3.5. Machine-Learning-Based Optimal DMR Selection and Disease Prediction

**Figure 5:**
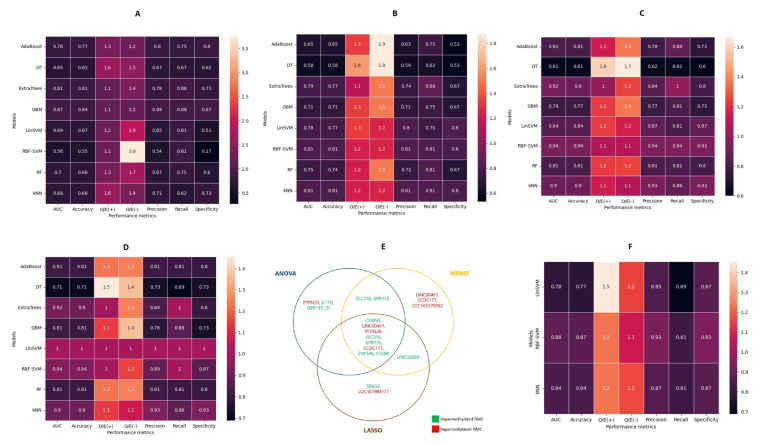
Performance of machine learning models for predicting OSCC using DMRs as features. (**A**) Initial models comprising all 105 DMRs. (**B**) Machine learning models based on ANOVA-selected DMR sets for predicting OSCC. (**C**) Machine learning models based on MRMR-selected DMR sets for predicting OSCC. (**D**) Machine learning models based on LASSO-selected DMR sets for predicting OSCC. (**E**) Selected DMR features by the three feature-selection methods and their concordance. (**F**) Machine learning models based on eight consensual DMRs for predicting OSCC.

CpG Methylation percentages along 100 bp regions were also used to generate models for OSCC diagnoses. The performance of the ML models based on all 105 DMRs are shown in Figure 5A, in which GBM has the best AUC, recall, and specificity of 0.87, 0.88, and 0.87, respectively. Fifteen DMRs were selected by ANOVA and MRMR, while 11 DMRs were obtained following LASSO selection. DMR sets selected by ANOVA performed worse than the models comprising all DMRs, in which RBF-SVM and kNN had a similar AUC, recall, and specificity of 0.81, 0.81, and 0.8 (Figure 5B). Of note, the DMRs selected by MRMR and LASSO had a better predictive performance for OSCC than those of ANOVA. The LASSO DMR set, irrespective of the ML algorithms, outperformed models based on the DMRs selected by ANOVA and MRMR (Figure 5B–D). Overall, linear SVM, based on the LASSO-selected DMRs, had the best performance of all models with a perfect AUC, recall, specificity, and calibration (1.00). Incorporating demographic variables to this out-performing methylome biomarker-based model did not affect its performance.

Eight DMRs were selected in all DMR sets according to the feature-selection techniques, and the genes associated with the regions are listed in Figure 5E and Table S2. The performance of the three outperforming models—RBF-SVM, linear SVM, and kNN—was assessed using this consensual DMR set and presented in Figure 5F. While the RBF-SVM model had a better classification accuracy than the kNN or linear SVM model (0.87 vs. 0.84 vs. 0.77), none of these consensual DMR models achieved the performance obtained for the optimal 11 DMRs selected using LASSO. Violin plots of the average SHAP values for the predicted classes of the LASSO-based linear SVM model are shown in Figure S5, in which the DMR associated with LINC00461 is the most important for OSCC stratification from OPMDs.

## 4. Discussion

Epigenetic alteration is a common event in oral carcinogenesis. Frequently, these deregulation signatures are characterized by global hypomethylation, involving the intergenic region, gene body, and repetitive sequences, as well as hypermethylation in the promoter regions [38]. Several aberrantly methylated genes and CpG islands implicated in oral cancer have been documented using an array of platforms that cover only about 3.1% of total CpG sites [39,40]. This has limited the discovery of novel methylome signatures for oral cancer beyond the confinement of conventional methylation arrays. What is more, these genome-wide techniques are infrequently applied to noninvasive samples which represent the clinical application of these biomarkers in real time. Recently, the feasibility of applying true whole-genome methylation-profiling methods, such as MethylCap-Seq, has been used with noninvasive samples for OSCC detection [41]; however, the method for operationalizing these sequencing-based methods has not been described [13]. Building on these lapses, this feasibility study utilized RRBS (which combines CpG enrichment and next-generation sequencing) to profile the salivary methylome, while optimizing the method with ML-based approaches for the detection of oral cancer for the first time.

The findings of this study confirmed that salivary methylome can be profiled both at the CpG site level and at a 100 bp regional level using genome-wide methods, such as RRBS, and it can be implemented with ML-based methods for OSCC diagnoses. CpG-based deregulation signatures were found to be robust, irrespective of different ML and feature-selection techniques than DMRs; however, the region-based signatures had a slightly higher recall in the comparison to the best-performing ML models based on both groups of biomarkers. The high discriminatory performance of both CpG sites and CpG regions in this study further supports similar studies that have reported on the use of genome-wide methylation approaches in other noninvasive (such as plasma) and tissue specimens for the diagnosis and prognosis of other malignancies [42,43,44]. Moreover, the findings of this study allude to the importance of comparing and combining different feature-selection techniques in the identification of robust biomarkers for cancer diagnoses. Of note, LASSO, an embedded selection technique, was found to be more robust in the selection of an optimal DMC/DMR set than univariate and multivariate methods of feature selection. Therefore, this study recommends the use of the technique to optimize the selection of CpG sites and regions from high-dimensional genome-wide methylation analysis; however, this may be performed while considering that excluded control variables deemed important may need to be added manually to the selected biomarkers [45,46]. However, variables such as age, gender, smoking and alcohol drinking status, as well as a family history of cancer, did not improve the performance of the best-performing models in this study.

While this study uniquely describes a comprehensive framework for performing the ML-assisted salivary methylome biomarker-based detection of OSCC, it is not without limitations. First, the salivary transcriptome was not profiled in this study and, as such, findings on the biological processes and pathways in which DMC and DMR-associated genes were involved needs to be confirmed in future reports. Such reports may assist in determining the ‘dynamic’ methylome profile of OSCC that is expressed in saliva in comparison to other oral mucosal diseases. Nonetheless, this did not represent the aims of this study and does not affect our main findings. Second, the optimal ML-based models using salivary DMCs/DMRs for oral cancer prediction would need to be validated in independent studies using the PRoBE protocol to determine their generalizability and the need for future optimization. The findings of such a study would also corroborate this study in designating the biomarkers as specific/preferential for OSCC because saliva represents a heterogeneous biofluid, and both normal and tumor cellular components contribute DNA for analysis. Third, we did not deliberately determine the human papillomavirus (HPV) infection status of the patients in our cohort. However, many OSCC patients were profiled to have HPV-negative tumors during histopathological diagnosis following tumor resection. It should also be noted that these investigations are not standard for patients with oral cancer or oral potentially malignant disorders but are mandatory for patients with oropharyngeal malignancies [47,48].

## 5. Conclusions

Overall, this study showed the feasibility of applying genome-wide methods of methylation analysis, such as RRBS, for biomarker discovery in oral cancer, which can be optimized further for ML-learning-based diagnoses. Both CpG-based and CpG-region-based biomarkers can be applied in the accurate diagnoses of oral cancer using feature-selection techniques, with LASSO being more robust across different ML classifiers than MRMR or ANOVA in this study. The linear SVM model based on 11 DMRs selected by LASSO had the best performance in discriminating between oral cancer and OPMDs in this study with perfect sensitivity and specificity, which suggests a potential clinical application following rigorous validation studies.

## Figures and Tables

**Table 1:** Characteristics of patients included for RRBS analysis.

Variables		OSCC (%)	OPMD (%)	Total	*p*-Value
Age	Median (IQR)	65 (57–72)	65.5 (53.8–72.8)	65 (57–72)	0.986 ^a^
Sex	Female	9 (52.9)	8 (50.0)	17 (51.5)	0.866 ^b^
	Male	8 (47.1)	8 (50.0)	16 (48.5)	
Site affected	Buccal	3 (17.6)	12 (75.0)	15 (45.5)	0.011 ^b^
	Palate	1 (5.9)	1 (6.3)	2 (6.1)	
	Tongue	8 (47.1)	3 (18.8)	11 (33.3)	
	Gingiva	5 (29.4)	0	5 (15.2)	
Risk habit category	NSND	11 (64.7)	7 (43.8)	18 (54.5)	0.227 ^b^
	SD	6 (35.3)	9 (56.3)	15 (45.5)	
Charlson comorbidity index	Median (IQR)	1 (0–2.5)	0	0 (0–1)	0.046 ^a^
Family history of cancer	Yes	4 (23.5)	4 (25.0)	8 (24.2)	0.922 ^b^
	No	13 (76.5)	12 (75.0)	25 (75.8)	
Hypertension	Yes	5 (29.4)	2 (12.5)	7 (21.2)	0.235 ^b^
	No	12 (70.6)	14 (87.5)	26 (78.8)	
Tumor stage	Stage I/II	6 (35.6)			
	Stage III/IV	11 (64.7)			
Tumor grade	Well differentiated	5 (29.4)			
	Moderately differentiated	9 (52.9)			
	Poorly differentiated	3 (17.6)			

^a^ Mann-Whitney U test; ^b^ Pearson’s Chi Square test/Fisher’s exact analysis.

## Data Availability

The data used in this study are not freely available due to agreed protection of patients’ epigenome data upon request for informed consent. However, anonymized data may be provided upon reasonable request by the corresponding author.

## Supplementary Materials

The following supporting information can be downloaded at: https://www.mdpi.com/article/10.3390/cancers14194935/s1, Table S1: List of optimal differentially methylated CpG sites selected by the feature-selection techniques; Table S2: List of optimal differentially methylated regions selected by the feature-selection techniques; Figure S1: Distribution of percent CpG site methylation for bases with at least 10× coverage in OSCC (A) and OPMDs (B) patients; Figure S2: Network plot of genes and their pathways enriched for DMC-associated genes; Figure S3: Violin plots for average SHAP values to explain the predicted outputs for ExtraTrees (A) and Linear SVM (B) models based on 6 DMC features; Figure S4: Gene ontology (GO-BP) (A) and KEGG pathway analysis (B) enrichment of genes associated with DMRs; Figure S5: Violin plot for average SHAP values to explain the predicted outputs for the optimal Linear SVM models based on 11 DMR features. File S1: Histogram plots of read coverage per base for all patients.
