# Supplementary material for: Machine Learning-Based Genome-Wide Salivary DNA Methylation Analysis for Identification of Noninvasive Biomarkers in Oral Cancer Diagnosis
Source: Cancers (Basel). 2022 Oct 8;14(19):4935. doi: 10.3390/cancers14194935 (PMC9563273; doi:10.3390/cancers14194935)

Table S1: List of optimal differentially methylated CpG sites selected by the feature selection techniques

CpG location	Status	Feature selection method			Associated genes
		ANOVA	MRMR	LASSO	
chr1.64472399	Hypermethylated	✓	✓		
chr2.185399968	Hypomethylated	✓		✓	
chr4.63036937	Hypermethylated			✓	
chr5.138214692	Hypermethylated	✓		✓	*CDC23*
chr5.164356723	Hypomethylated		✓		
chr6.12749625	Hypermethylated	✓	✓		
chr6.378710	Hypomethylated		✓		*IRF4*
chr6.101444040	Hypermethylated		✓		
chr7.125891874	Hypomethylated		✓		
chr7.149925393	Hypomethylated			✓	
chr7.125918264	Hypomethylated			✓	
chr8.143404014	Hypomethylated	✓	✓		
chr8.98757391	Hypomethylated			✓	
chr10.6889479	Hypomethylated	✓	✓	✓	
chr11.69766784	Hypomethylated	✓	✓	✓	*FGF4*
chr15.24904609	Hypomethylated	✓			*SNRPN*
chr17.80594125	Hypomethylated	✓			
chr17.67245746	Hypermethylated		✓		*HELZ*
chr19.42391067	Hypomethylated	✓	✓	✓	*CNFN*
chr19.48929535	Hypomethylated	✓	✓	✓	*DHDH*
chr19.58446797	Hypomethylated	✓			*ZNF324B*
chr21.9839525	Hypermethylated	✓	✓	✓	*LINC01667*
chr21.9839531	Hypomethylated			✓	*LINC01667*
chr22.10741490	Hypomethylated	✓	✓	✓	
chr22.38803332	Hypermethylated	✓	✓		*DNAL4*

Table S2: List of optimal differentially methylated regions selected by the feature selection techniques

**Region location**			**Status**	**Feature selection method**			**Associated genes**
Chromosome	Start	End		ANOVA	MRMR	LASSO	
chr1	159900126	159900225	Hypermethylated	✓	✓	✓	*CFAP45*
chr5	88672901	88673000	Hypomethylated	✓	✓	✓	*LINC00461*
chr5	88672876	88672975	Hypomethylated		✓		*LINC00461*
chr10	22345801	22345900	Hypermethylated			✓	*SPAG6*
chr10	46911301	46911400	Hypomethylated	✓	✓	✓	*PTPN20*
chr10	46911326	46911425	Hypomethylated	✓			*PTPN20*
chr10	100519876	100519975	Hypermethylated	✓	✓	✓	*SEC31B*
chr10	100519901	100520000	Hypermethylated	✓	✓		*SEC31B*
chr11	115660251	115660350	Hypermethylated		✓	✓	*LINC02698*
chr13	20721476	20721575	Hypermethylated	✓			*IL17D*
chr14	59464176	59464275	Hypermethylated	✓			*GPR135*
chr14	59464201	59464300	Hypermethylated	✓	✓		*GPR135*
chr14	59464226	59464325	Hypermethylated	✓			*GPR135*
chr14	59464251	59464350	Hypermethylated	✓	✓	✓	*GPR135*
chr14	69571751	69571850	Hypomethylated	✓	✓	✓	*CCDC177*
chr14	69571776	69571875	Hypomethylated		✓		*CCDC177*
chr15	93280651	93280750	Hypomethylated		✓		
chr15	89378976	89379075	Hypomethylated			✓	
chr19	30375351	30375450	Hypermethylated	✓	✓	✓	*ZNF536*
chr20	30922301	30922400	Hypermethylated	✓	✓	✓	*FCGBP*
chr22	46536651	46536750	Hypermethylated	✓	✓		*CELSR1*

Figure S1: Distribution of percent CpG site methylation for bases with at least 10X coverage in OCSCC (A) and OPMDs (B) patients.

A **B**

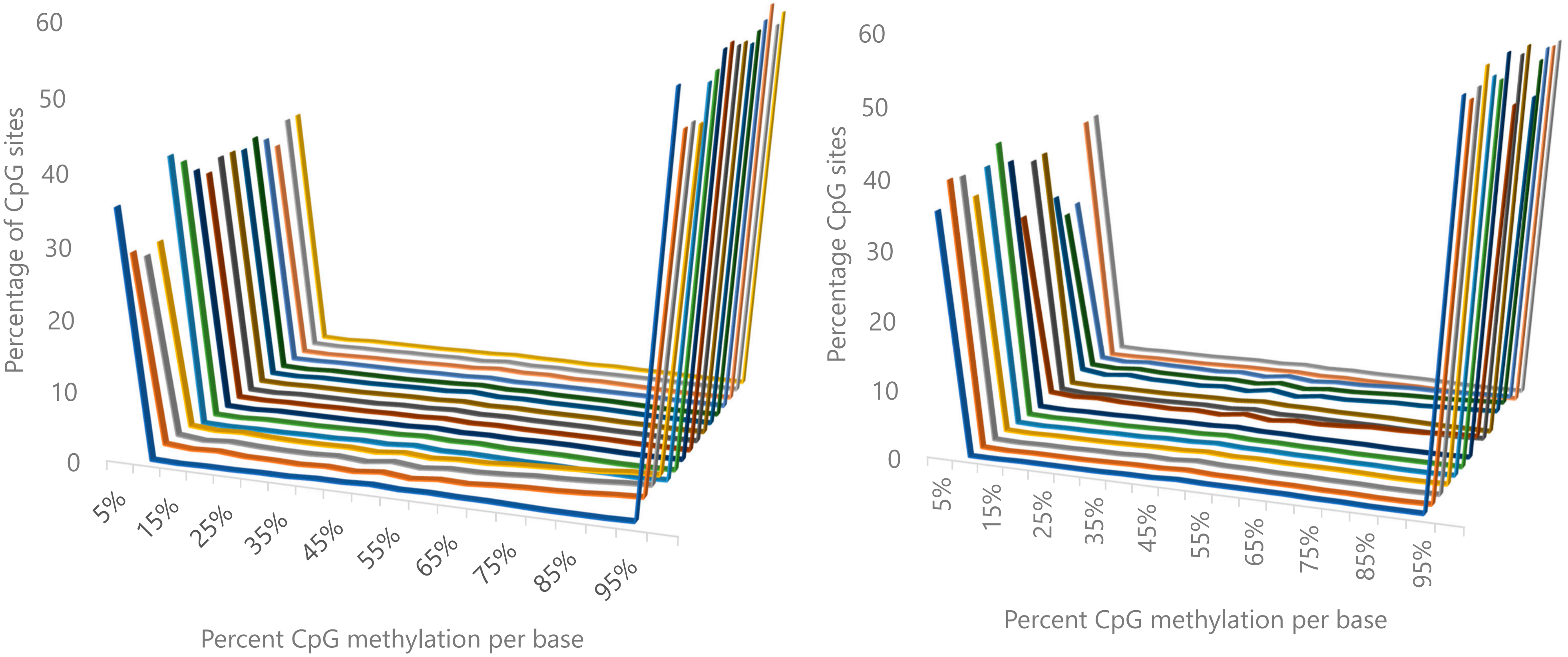

Figure S2: Network plot of genes and their pathways enriched for DMC-associated genes.

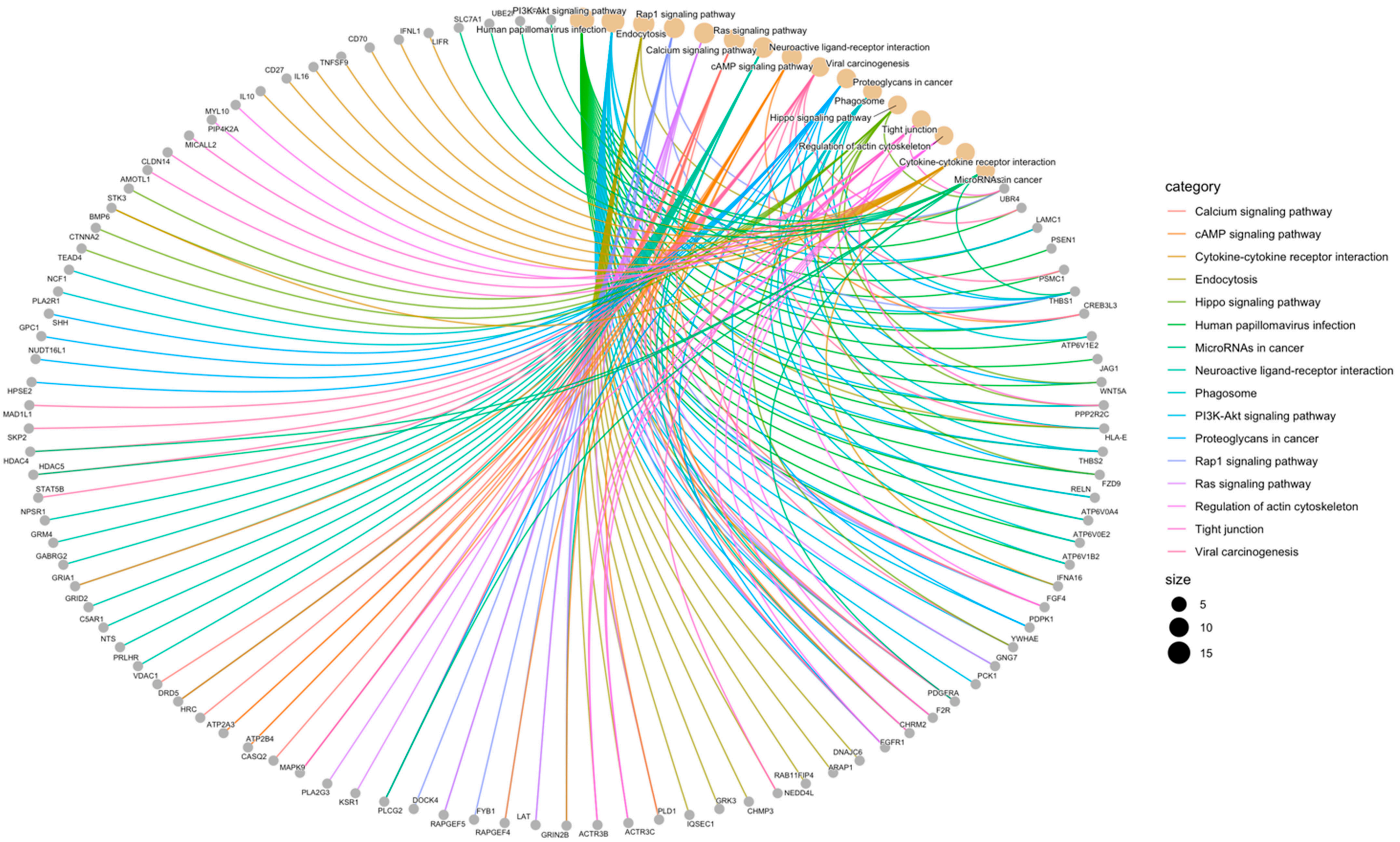

Figure S3: Violin plots for average SHAP values to explain the predicted outputs for ExtraTrees (A) and Linear SVM (B) models based on 6 DMC features.

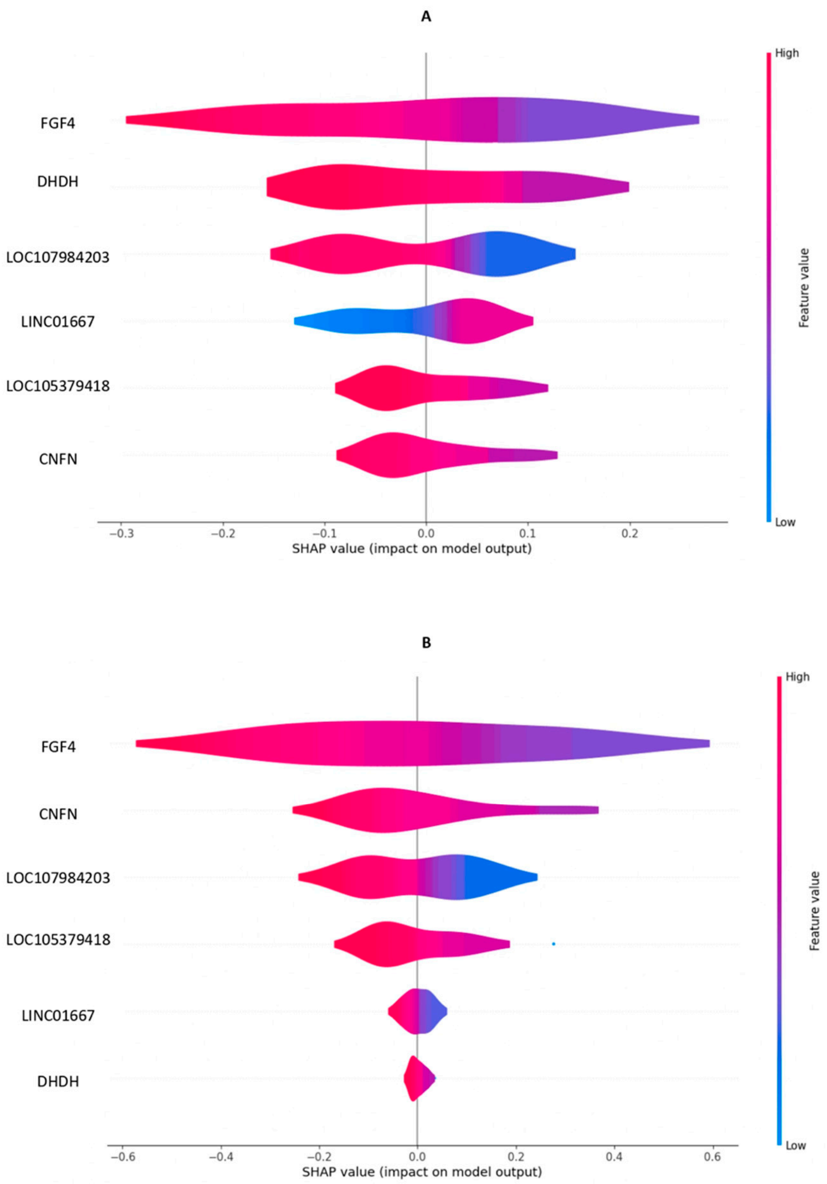

Figure S4: Gene ontology (GO-BP) (A) and KEGG pathway analysis (B) enrichment of genes associated with DMRs.

A

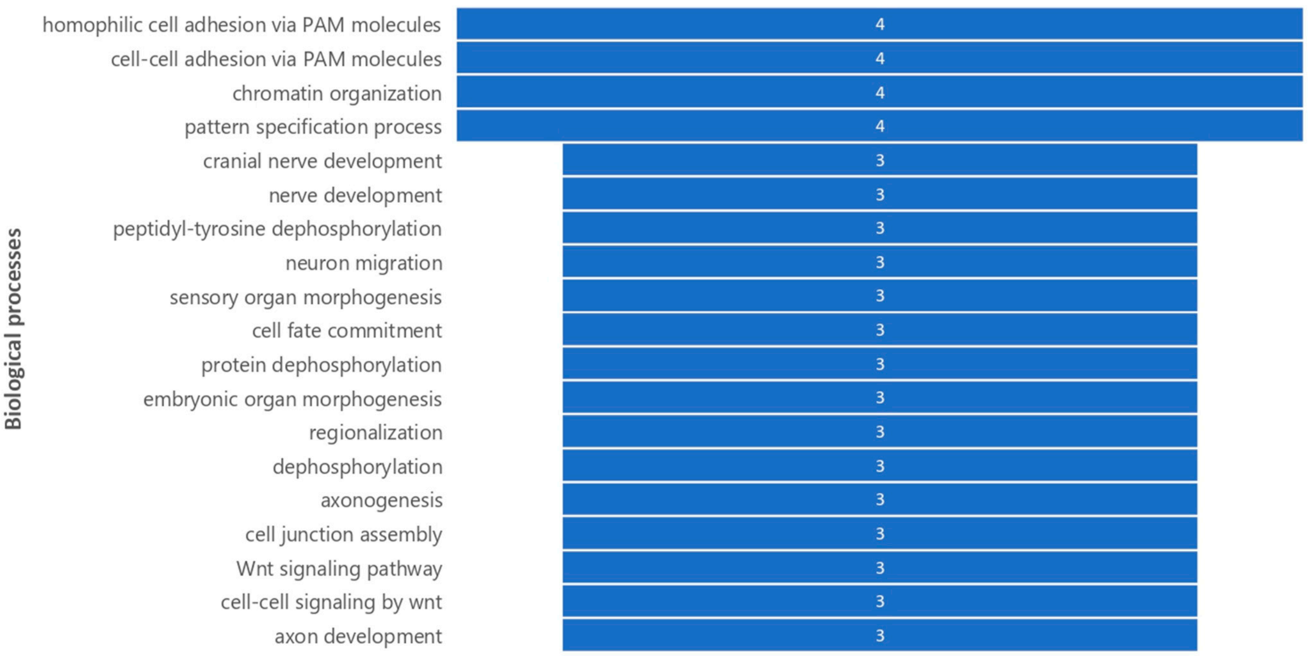

B

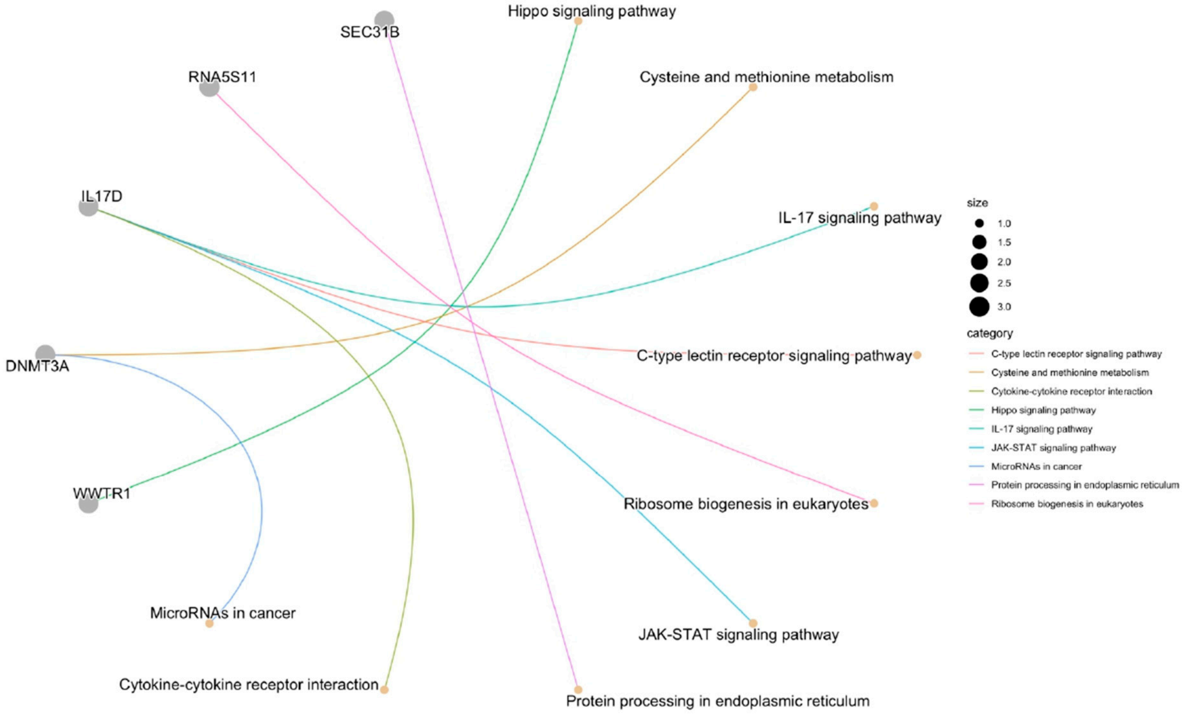

Figure S5: Violin plot for average SHAP values to explain the predicted outputs for the optimal Linear SVM models based on 11 DMR features.

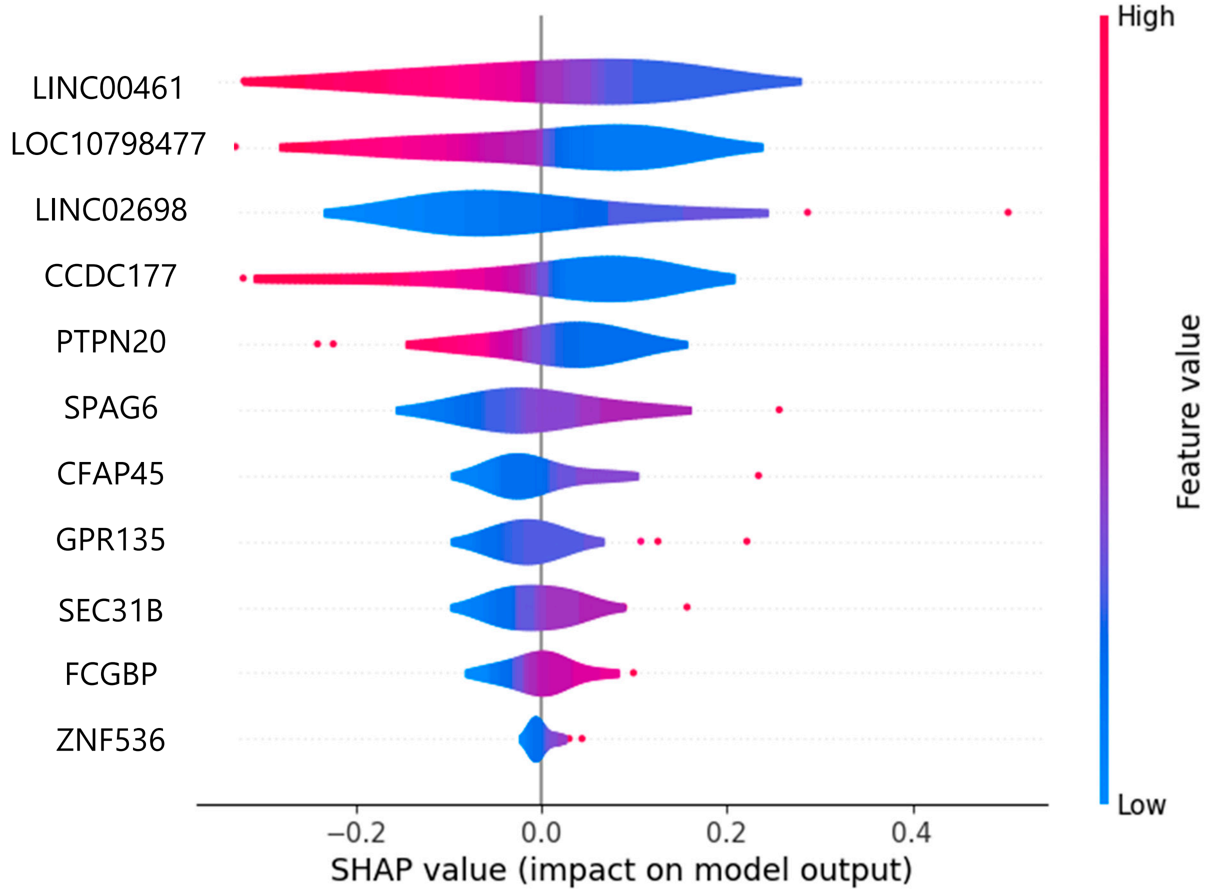

Supplement: Supplementary file 1 [file cancers-14-04935-s001.zip › Supplementary figures and tables.pdf]
